# Milk Fatty Acid Profiles in Different Animal Species: Focus on the Potential Effect of Selected PUFAs on Metabolism and Brain Functions

**DOI:** 10.3390/nu13041111

**Published:** 2021-03-28

**Authors:** Maria P. Mollica, Giovanna Trinchese, Fabiano Cimmino, Eduardo Penna, Gina Cavaliere, Raffaella Tudisco, Nadia Musco, Claudia Manca, Angela Catapano, Marcellino Monda, Paolo Bergamo, Sebastiano Banni, Federico Infascelli, Pietro Lombardi, Marianna Crispino

**Affiliations:** 1 Department of Biology, University of Naples Federico II, 80126 Naples, Italy; mariapia.mollica@unina.it (M.P.M.); giovanna.trinchese@unina.it (G.T.); fabiano.cimmino@unina.it (F.C.); eduardo.penna@unina.it (E.P.); gina.cavaliere@unina.it (G.C.); angelacatapano@me.com (A.C.); crispino@unina.it (M.C.); 2 BAT Center—Interuniversity Center for Studies on Bioinspired Agro-Environmental Technology, University of Naples ‘Federico II’, 80055 Naples, Italy; 3 Department of Veterinary Medicine and Animal Production, University of Napoli Federico II, 80100 Naples, Italy; tudisco@unina.it (R.T.); nadia.musco@unina.it (N.M.); infascel@unina.it (F.I.); pilombar@unina.it (P.L.); 4 Department of Biomedical Sciences, University of Cagliari, Monserrato, 09042 Cagliari, Italy; claumanca@unica.it (C.M.); banni@unica.it (S.B.); 5 Department of Pharmacy, University of Naples Federico II, 80131 Naples, Italy; 6 Department of Experimental Medicine, Section of Human Physiology and Unit of Dietetics and Sports Medicine, Università degli Studi della Campania “Luigi Vanvitelli”, 80138 Naples, Italy; marcellino.monda@unicampania.it; 7 Institute of Food Sciences, National Research Council, 83100 Avellino, Italy

**Keywords:** human health, milk, fatty acids, animal feeding, brain functions, omega-3 PUFAs, omega-6 PUFAs, CLA

## Abstract

Milk contains several important nutrients that are beneficial for human health. This review considers the nutritional qualities of essential fatty acids (FAs), especially omega-3 (ω-3) and omega-6 (ω-6) polyunsaturated fatty acids (PUFAs) present in milk from ruminant and non-ruminant species. In particular, the impact of milk fatty acids on metabolism is discussed, including its effects on the central nervous system. In addition, we presented data indicating how animal feeding—the main way to modify milk fat composition—may have a potential impact on human health, and how rearing and feeding systems strongly affect milk quality within the same animal species. Finally, we have presented the results of in vivo studies aimed at supporting the beneficial effects of milk FA intake in animal models, and the factors limiting their transferability to humans were discussed.

## 1. Introduction

In recent years, awareness of the importance of diet for human health has considerably increased, and some consumers, mainly living in developed countries, are paying more attention to selecting food that may directly contribute to their health [1]. Thus, food is no longer intended only to satisfy hunger and to provide the necessary nutrients, but also to prevent metabolic diseases and to improve the wellness of consumers [2].

Healthy nutrition, together with physical activity, positively affects metabolic health, the immune system, and lowers the risk of chronic illnesses and infectious diseases [3]. A variety of selected nutrients such as vitamins, minerals, dietary fibers, proteins, antioxidants, and fatty acids (FAs), but also drinking enough water and limiting sugar, fat, and salt, significantly lower the risk of several pathologies such as overweightness, obesity, heart disease, stroke, diabetes, neuro-pathologies, and certain types of cancer [4].

Common dietary recommendations for human health suggest limiting the consumption of several foods of animal origin [4] and, in general, to reduce saturated fatty acids (SFAs) and trans fatty acids to lower the incidence of cardiovascular disease [5]. Nonetheless, many studies demonstrated no association between the consumption of full-fat dairy and the risk of cardiometabolic diseases [6]. In this regard, milk deserves a dedicated discussion.

Milk is consumed by over 6 billion people around the world. Fats from milk and dairy products are an important source of nutrients and energy, but they have been reported as being implicated in some chronic diseases in humans [7]. Indeed, it is commonly suggested to use low-fat or reduced-fat products [8]. Nonetheless, recent studies have shown that milk and dairy, regardless of their fat content, may have a beneficial effect on metabolic and cardiometabolic health [9,10]. With regard to the effects of milk on brain health, although some studies indicated a positive impact of dairy consumption on cognitive performance and in reducing the risk of dementia [11,12], most of the results are still controversial [13,14,15]. In particular, little is known about the mechanisms linking milk/dairy intake and cognitive performance, and, in general, there is a lack of knowledge of the efficacy of individual milk nutrients on the central nervous system (CNS). Therefore, our review will focus on the effects of some FAs present in milk on human health, including the impact on the CNS.

It is important to underline that the term “milk” is vague since different animal species yield milk of different quality, with different compositions of FAs. Interestingly, the particular species-specific milk composition, especially protein and fat content, determine the rate of growth of the offspring of each mammalian species [16]. In addition, within the same animal species, and even within the same breed, the quality of milk strongly depends on several factors, such as animal feeding, rearing systems, and seasonal variability. Thus, in our review we will analyze the differences in the FA profiles of milk produced by different animal species, and by animals reared and fed in different ways. 

## 2. Profile of Milk Fat

Milk contains numerous important nutrients such as FAs (oleic acid, ω-3 FAs), protein, lactose, mineral salts (calcium, phosphorus, potassium, magnesium, sodium), and vitamins (especially B1, B2, B6, retinol, carotenes, tocopherol). The FA profile of milk is an important factor in determining its nutritional value [10,17], with special attention paid to essential FAs, namely linoleic acid (LA, C18:2 ω-6) and α-linolenic acid (ALA, C18:3 ω-3), which contribute to the human daily intake of polyunsaturated fatty acids (PUFAs). These FAs cannot be synthesized in humans [18].

### Polyunsaturated Fatty Acids (PUFAs)

FAs are important components of the human body, playing several structural and functional roles. They are not only an important source of energy, but they are also key components of cell membranes’ phospholipids, contributing to the fluidity, flexibility, and permeability of the membrane [19]. Key FAs are ω-3 PUFAs, which participate in the inflammatory cascade, reducing the oxidative stress and playing a protective role in the cardiovascular and nervous systems [20,21]. The ALA in normal conditions cannot be converted efficiently to more biologically active PUFAs such as very long-chain eicosapentaenoic acid (EPA), docosahexaenoic acid (DHA), and docosapentaenoic acid (DPA, 22:5 ω-3) [22], which have to be assumed by diet. Milk and dairy products contribute about 16–26% of the human daily intake of ω-3 PUFAs [23].

The main ω-6 FA in cow milk fat, LA, is required for the synthesis of arachidonic acid (AA, C20:4, ω-6) and eicosanoids [24]. The term conjugated linoleic acid (CLA) indicates a group of LA isomers with conjugated double bonds, produced in the rumen as well as in the mammary gland by Δ9–desaturase activity [25,26,27,28]. In particular, CLA refers to two isomers: the cis-9, trans-11 (c9, t11; also known as rumenic acid, RA) and, at a much lower concentration, the trans-10, cis-12 (t10, c12) isomer, which are mainly present in the milk of ruminant animals [17]. RA is the main CLA component, accounting for 90% of the total CLA in ruminant fat [29], but a range of cis–trans or trans–trans CLA isomers with the trans bond at C-7 to C-14 and the cis bond at C-9 to C-13 are also found. CLA isomers are naturally produced in the rumen as intermediates of the gut bacterial biohydrogenation of dietary LA and ALA, present in fresh grass, to stearic acid [30]. Alternatively, they may originate from endogenous synthesis in the mammary gland by the action of stearoyl-CoA desaturase on trans11 C18:1 (trans-vaccenic acid, tVA) [31].

## 3. Milk Fatty Acid Composition in Different Animal Species

Milk for human nutrition produced worldwide mainly comes from cows (about 85%), followed by buffalo (11%), goat (2.3%), sheep (1.4%), and camel (0.2%), while milk from other dairy species such as horse, donkey, and yak, accounts for less than 0.1% [32].

### 3.1. Ruminants Milk

The milk of ruminants is characterized by a high content of SFAs (about 70% of total FAs), a low content of PUFAs (less than 3%, including ω-3 FAs and CLA, mainly RA), and trans-FAs (~4%, including vaccenic acid or trans-11 C18:1) [33,34]. There are relevant differences between species (Table 1): milk fat content is higher in sheep (6–7 g/100 g of milk) than in cows (3.5–3.8 g/100 g of milk) or goats (3.6–4 g/100 g of milk) [35]. In addition, the fat of milk from sheep and goats contains high levels of short- and medium-chain FAs with a carbon chain consisting, respectively, of 4–10 or 11–15 carbon atoms. In fact, the FAs known as caproic (C6:0), caprylic (C8:0), and capric (C10:0) are typical of goat milk and represent up to 15–18% of the total milk FAs, compared to 5–9% of cow’s milk [36], which also helps to improve milk digestibility. In cow milk fat the main ω-6 FA is LA, which is required for the synthesis of AA (C20:4, ω-6) and eicosanoids [24].

Buffalo milk contains more than twice the amount of fat (7.5–8.7 vs. 3.5–3.8 g/100 g milk) than cow milk, resulting in higher energy content. The percentage of SFAs (65–75 g/100 g of total FAs) is comparable to cow milk. The high fat content makes buffalo milk particularly suitable for the production of dairy products (e.g., mozzarella cheese, a typical fresh and stringy-textured cheese) [43].

Goat milk is recommended for consumers who are allergic to cow milk. It contains high levels of butyric (C4:0), caproic (C6:0), caprylic (C8:0), capric (C10:0), lauric (C12:0), myristic (C14:0), palmitic (C16:0), and linoleic (C18:2) acids, but low levels of stearic (C18:0) and oleic (C18:1) acids. At least 20% of the FAs in goat milk are short chain FAs, which are readily digested [44], and the content of medium-chain FAs is relatively high [45].

Sheep milk shows significantly higher levels of caproic (C6:0), caprylic (C8:0), capric (C10:0), and lauric (C12:0) acids than cow milk [46]. These short- and medium-chain FAs are associated with the characteristic flavors of cheeses. In addition, sheep milk is characterized by a higher concentration of butyric acid (C4:0) and ω-3 FAs than other ruminant milk [47].

### 3.2. Non-Ruminants (Monogastric Animals) Milk

Equid milk is characterized by a total fat content (0.8–1.5%) much lower than human milk (3.5–4.2%) and a unique lipid composition. The FA composition and, in particular, the percentage of total PUFAs of horse and donkey milk are high. The ω-3 PUFA content averages 8.66–11.97% and 9.45–9.64% of total FAs for horse and donkey milk, respectively. Furthermore, eicosapentaenoic acid (C20:5 ω-3), DHA (C22:6 ω-3), and AA (C20:4 ω-6) levels in horse and donkey milk are below 0.5 g/100 g FAs [48,49].

Donkey and horse milk have been used in human nutrition since ancient times, in particular as a cow milk substitute for allergic/intolerant people and elderly consumers [49], and as foods with health-promoting properties related to their peculiar lipid composition and lactose contents [50]. It is noteworthy that donkey and human milk are both characterized by a peculiarly high concentration of palmitic acid in the sn-2 position of the triacylglycerol backbone that seems to play a crucial role in the regulation of energy and lipid metabolism [51]. Accordingly, the administration of donkey and human milk to rats displayed several beneficial effects on lipid and glucose metabolism compared to animals fed cow milk [37,52,53,54]. It has been hypothesized that this effect depends on the modulation of oleoylethanolamide (OEA) and palmitoylethanolamide (PEA) levels [55], an endogenous ligand of the nuclear peroxisome proliferators-activated receptor (PPAR)-α, which regulates fatty acid metabolism [56,57].

### 3.3. Human Milk

It is noteworthy that the primary source of the nutrients and lipids, including monounsaturated (MUFAs), ω-6, and ω-3 FAs, necessary for human development is maternal milk [58,59]. Since humans are unable to synthesize ω-6 or ω-3 FAs, all the fatty acids secreted into human milk are derived from the maternal diet. Thus, differences in maternal lipid nutrition during lactation can result in large differences in ω-6 and ω-3 FAs in human milk [60].

In human milk, the amount of fat (3.8–4.2%) is similar to cow milk, while the composition of FAs is very different from cow milk and more similar to donkey milk. Indeed, human milk fat is high in unsaturated fatty acids and PUFAs, particularly the essential fatty acids LA and ALA. The beneficial effects of human milk also depend on its bioactive/immunomodulatory factors (oligosaccharides, lactose, glycosaminoglycans) [61]. The World Health Organization recommends the exclusive breastfeeding of infants for the first 6 months of age, since maternal milk reduces the incidence of neonatal necrotizing enterocolitis, gastroenteritis, respiratory infection, and immunologically based disease, and improves later cognitive development [62,63].

## 4. Factors Affecting Fatty Acid Profiles in Milk from Different Animal Species

Dairy research initially focused on increasing milk yield and the production efficiency of cows. During the past few decades, consumers and industry demand for healthy and sustainable foods motivated the great research efforts aimed at investigating the factors influencing the nutritional quality of the final product. Animal milk composition is affected by several factors (animal breed, health status, stage of lactation, feeding regimen, and seasons), and among them, animal diet was recognized as an effective way to maximize the content of beneficial FAs in milk (ω-3 PUFAs, tVA, and RA) [64]. Manipulating the diet of cows, as well as of goats and sheep, was an effective way to increase the health-promoting FA content of milk [47,65]. Accordingly, the beneficial effect of the feeding practices used in organic husbandry on cow, goat, and sheep milk FA composition was demonstrated [66,67,68,69,70].

As demonstrated in sheep, the higher levels of ALA in milk from the grazing group were due to the higher content of ALA in the pasture, as opposed to in alfalfa hay [71]. Thus, grazing is a useful tool for increasing the levels of milk PUFAs [72], as shown in sheep, dairy cows, dairy buffalos, and dairy goats [69,73,74,75,76].

In addition, it was reported that the highest PUFA values in the milk of the grazing group occurred in June and September, in accordance with the pasture’s fatty acid profile during the trial [77,78]. A similar result was also reported in grazing sheep [79,80,81]. The higher levels of c9, t11 CLA and t10, c12 CLA detected in the milk from grazing animals could be also due to the higher levels of LA and ALA, the main CLA precursors, which were higher in the pasture than in the hay [82]. Indeed, according to Aii et al. [83], the hay making process determines a loss in these fatty acids. Thus, the pasture, especially when rich in ALA, represents a good strategy for improving the lipid composition of milk in ruminant species. In addition, grazing, or at least using fresh forage, may represent a low-cost approach when compared to supplementing the diet with oilseeds or fats [84]. According to Benbrook et al. [85], a significant decrease of LA, higher levels of ALA, long-chain ω-3 FAs, total CLA, and a ω-6/ω-3 ratio close to 1 can be achieved in milk by increasing the forage portion of the diet of dairy cows. Finally, Davis et al. [86] reported a 94% and 92% increase in CLA and ω-3 FA, respectively, as well as a significant decrease in the ω-6/ω-3 ratio in milk from grazing dairy cows.

It is noteworthy that dairy products’ FA profiles mainly depend on raw milk content, rather than cheese processing methods [27,87,88]. Sheep cheese has higher levels of beneficial PUFAs than cow and goat cheese, making the improvement of FA profiles in this cheese particularly interesting from a nutritional point of view [88]. Cheese fats produced by milk from grazing animals or animals fed high amounts of fresh forage have higher proportions of ω-3 FAs vs. conventional cheese because forages are naturally rich sources of C18:3 ω-3 [89,90]. Additionally, higher levels of PUFAs and MUFAs, lower percentages of SFAs, higher content of C18:3 and CLA, and a better atherogenic index value have been reported for buffalo mozzarella cheese when the animals were fed fresh forage [91].

Ruminants’ diet may also affect the milk concentration of the other two classes of bioactive FAs: (i) the branched-chain fatty acids (BCFAs), characterized by anti-tumor activity [92] and the ability to decrease the incidence of necrotizing enterocolitis [93] and to support pancreatic β-cell function [94]; and (ii) the odd-chain fatty acids (OCFAs), which reduce the risk of type-2 diabetes [95,96] and cardiovascular diseases [96,97]. The main source of odd- and branched-chain fatty acids (OBCFAs) in the human diet is represented by ruminant fats. Indeed, they are synthesized de novo by the rumen microbial flora [98], but linear OCFAs (C15:0 and C17:0) and anteiso-isomers can be also produced in the mammary gland [98,99]. These multiple origins could explain their higher concentration than other OBCFAs in goat [100] and cow milk [93,101,102]. Lopez et al. [103] reported significantly higher OBCFA concentration in organic goat (4.7%) than in conventional milk (3.4%). Significantly higher levels of total OBCFAs are reported in milk from goats fed a diet with a high, rather than low, forage:concentrate ratio [103,104,105]. This high level may be due to a better equilibrium in the rumen bacterial populations with the administration of higher amounts of forage. In goats, milk OBCFA content was also significantly influenced by the diet lipid supplementation [106].

In this animal species, milk OBCFA content was also significantly influenced by the diet lipid supplementation and the forage:concentrate ratio [104,105,106]. Accordingly, Vlaeminck et al. [98] observed a decrease in milk OBCFAs in cows by increasing their dietary concentrate percentage from 20 to 70% [98]. In addition, milk from cows fed different pasture vegetation types showed significantly different contents of OCFAs (C15:0, C17:0, C17:1) [107].

## 5. Polyunsaturated Fatty Acids Effects on Human Health

### 5.1. Polyunsaturated Fatty Acids Effects on Metabolism

It is well known that a deficiency of ω-3 FAs is detrimental for human health [108]. The practical way to increase levels of DPA, EPA, and DHA in the body does not depend exclusively on ω3 processing, but requires their intake directly from foods and/or dietary supplements. Indeed, the most recent dietary guidelines recommend the consumption of foods containing high levels of ω-3 PUFAs. ω-3 PUFAs exert beneficial effects [109] in lowering the risk of cardiovascular disease [110], in reducing the advancement of the atherosclerotic process in patients with coronary heart disease [20], in slowing down the growth of cancer, and in increasing the efficacy of chemotherapeutic treatments [111], as well as in lowering neuroinflammation and maintaining mental health [21]. In addition, DHA and EPA are considered potent anti-inflammatory products, able to reduce the risk of insulin resistance and ameliorate obesity-associated disorders [112,113]. Interestingly, in a rodent model with a high-fat diet regimen, it was demonstrated that the replacement of lard (rich in SFAs) with fish oil (rich in ω-3 PUFAs) limited the development of systemic and tissue inflammation, reduced fat accumulation in the liver and skeletal muscle, attenuated insulin resistance and oxidative stress, and modulated mitochondrial efficiency [114,115]. Limited information is available on the biological effects of DPA in clinical studies, since pure DPA has become commercially available only recently.

The synthetic isomeric mixture (RA:t10, c12, 1:1) of CLA has a broad range of beneficial effects on inflammation, obesity, diabetes, and cancer [116,117]. It has been known for a long time that CLA isomers have distinct effects, although there is no complete agreement on their specific effects on sugar/lipid metabolism and anticancer activity. In particular, the pro-diabetic effect of t10, c12-CLA was initially demonstrated in obese subjects [118], while the anti-diabetic effects of RA were indicated by other studies [119,120,121]. Recently, the anti-inflammatory efficacy of RA was reported, while t10, c12-CLA was indicated as responsible for anti-carcinogenic, anti-obesity, and anti-diabetic effects [122]. In rodent models, it was demonstrated that RA had the ability to improve antioxidant detoxifying defences via the activation of the nuclear erythroid-related factor 2 (Nrf2)—which is the main pathway in the maintenance of redox status and metabolic and protein homeostasis, as well as in the regulation of inflammation [123,124,125]. In addition, it has been recently shown that CLA, by inducing PPAR-α, greatly improves DHA biosynthesis from ALA in animal models [126] and in humans [127].

Interestingly, it was demonstrated that the milk obtained from cows treated with a different feeding/rearing system exhibited a different profile of milk PUFAs and had selective metabolic effects when administered to rodents. In particular, rats’ diets were supplemented with milk produced by dairy cows fed with a high forage:concentrate ratio (70:30; high-forage milk), and with milk produced by cows fed with the low forage:concentrate ratio (55:45; low-forage milk) normally used in intensive farms. The high-forage milk had beneficial effects on the rats’ lipid metabolism, inflammation, and oxidative stress, modulating mitochondrial function and efficiency in the liver and skeletal muscle. These data provided the first evidence that dietary supplementation with high-forage milk in rodents decreases lipid accumulation through an increase in fatty acid oxidation, and a decrease in inflammation and oxidative stress [37,128]. The beneficial effects of high-forage milk were similar to those resulting from ω-3 and RA intake [114,115,129,130], allowing the authors to hypothesize that ω-3 and RA may be some of the key functional components of high-forage milk.

In an another set of experiments, mice were fed with high-fat diets containing either dairy cream obtained from the milk of cows fed on pastures, or standard cream. It was observed that the animals fed pasture dairy cream exhibited improved intestinal barriers, and this effect was attributed to the higher LA content of pasture dairy cream [131]. On the other hand, when mice models with chemically induced colitis were fed cow milk naturally enriched in RA (selected from commercially available organic blends), high cyto-protective effects were observed, presumably associated with RA’s ability to trigger Nrf2-mediated antioxidant/detoxifying defenses [132].

The potential health benefits resulting from CLA intake, mainly obtained in animal models, led to its recognition as a functional food [133]. In clinical studies, the health effects produced by the intake of CLA-rich dairy products from goat or sheep milk were evaluated [127,134,135]. Since high CLA concentration improves the nutritional value of dairy foods, the production of dairy food enriched with RA was encouraged [136,137]. Surprisingly, despite the great number of studies that investigated the factors determining the concentration of health-promoting FAs in milk, only a limited number of analyses have been focused on the biological effects produced by the intake of RA-rich dairy products (Table 2). The main limitation of most of these studies is due to the inadequate evaluation of the role of RA on the observed effects, and the lack of conversion of the RA-enriched supplement into doses that can be, reasonably, consumed by humans [127,134,138]. Interestingly, the intake of dairy products naturally enriched in RA and tVA (organically produced) was demonstrated to increase the content of these FAs in the milk of lactating women [139,140,141]. On the other hand, results related to the protective effect of RA-rich dairies against breast cancer are still controversial [128,129].

Taken together, these results indicate that, although nutritional benefits may potentially be provided by the intake of dairy foods with improved RA content, additional investigation with multi-center clinical trials is necessary to identify the population more responsive to them.

### 5.2. Polyunsaturated Fatty Acids Effects on Nervous System

The brain requires an outstanding energy supply to maintain its dynamic abilities and its complex architecture. Indeed, the adult brain, which accounts for about 2% of body weight, utilizes 20% of the oxygen and 20–25% of the glucose [151]. In addition, the huge brain size expansion during the first few years of life requires specific nutrients, such as lipids, proteins, and micronutrients, which are also necessary to support neuronal connectivity and brain cognitive functions in adulthood. Thus, the appropriate nutritional management of the brain has a crucial relevance throughout the whole life [152]. It has been widely recognized that dietary regimen affects brain health and the appropriate choice of diet can reduce the risk of mental illness. Although no single dietary component has been identified as crucial in improving brain wellness, eating habits and the synergistic interaction between different nutrients seem to have beneficial effects in preventing mental health disorders [153]. Thus, diet and its bioactive components are among the modifiable risk factors possibly influencing the development of neuro-pathologies. In particular, SFAs and simple carbohydrates seem detrimental for the brain, while PUFAs, polyphenols, and antioxidants appear to be neuroprotective [154,155,156].

The brain is the second organ richest in lipids after adipose tissue, with 35% of brain lipids made by PUFAs. The biosynthesis of PUFAs in the brain is very low, and most of them are biosynthesized in the liver, although the contribution of diet is also critical and mainly dependent on fish consumption, which is often under the recommended daily intake [157,158,159]. The most abundant PUFAs in the brain are ω-6 AA and ω-3 DHA, which are components of plasma membranes’ phospholipids in neuronal cells [21]. ω-3 DHA is indispensable in maintaining membrane integrity and, as a consequence, membrane ionic permeability, neuronal excitability, mitochondrial activity, and synaptic functions. Thus, DHA is crucial for the normal development of the CNS, and its deficiency can impair cerebral functions, leading to neuropsychiatric disorders such as depression or dementia [159]. Animal models with a chronic deficiency of dietary ω-3 PUFAs, from conception to the early stage of development, exhibited a decreased concentration of DHA in neuron membranes, leading to retarded visual acuity, impaired learning ability, and several neurological disorders [21,157]. Since having the correct supply of ω-3 PUFAs during development is so important for brain activity, the fetus receives the necessary PUFAs through the placenta, and the newborn receives them by breast-feeding [21]. This is one of the reasons for highly recommend breast-feeding for infant nutrition. Since breastfeeding may not always be possible or suitable, infant formula is the industrial substitute for newborn nutrition [160]. In this regard, it has been demonstrated that adding DHA to formulas for bottle-fed human infants increases their blood concentration of DHA to values similar to breast-feeding infants and significantly improves mental development [157]. The importance of the correct supply of ω-3 PUFAs during the brain development was also demonstrated using an interesting experimental paradigm. Rats were exposed to a diet deficient in ω-3 FAs during the brain maturation period. When they became adults, their diet was switched to a Western diet, and they were subjected to mild traumatic brain injury, a risk factor for the development of psychiatric illness. In the animals exposed to this diet switch, compared to controls, the mild brain injury induced more severe anxiety-induced disorders, and the downregulation of the brain’s levels of brain derived neurotropic factor (BDNF) and its signalling pathway was observed [161]. These data underlined how incorrect dietary habits during development might lower the threshold for neurological disorders later in life. Interestingly, patients with depression display lower levels of ω-3 PUFAs [162] and decreased serum levels of BDNF [163]. Accordingly, the administration of ω-3 PUFAs stimulated BDNF expression and adult hippocampal neurogenesis in mice [164,165,166].

Since BDNF is a protein known to couple energy metabolism and synaptic plasticity [158], it is possible to suggest that dietary PUFA is acting as an energy modulator in cognitive functions, affecting, in particular, the synaptic regions [167]. From this point of view, it is interesting to underline that some neurodegenerative diseases, as well as aging processes, share, as a common feature, synaptic dysfunctions, and therefore are defined as “synaptopathies” [168,169,170,171,172]. Interestingly, PUFAs play critical roles at the synapses, where they are involved in synaptogenesis, synapse maintenance, and synapse function [168]. Thus, understanding PUFAs’ action mechanisms at synaptic areas will open the way to an innovative and promising strategy to treat synaptopathies using PUFA dietary supplementation which may restore the impaired synaptic functions [168]. Recently, using a very elegant experimental approach, it was demonstrated that DHA is able to mitigate the presynaptic over-inhibition of animal model autism spectrum disorders [173].

Interestingly, ω-6 and ω-3FAs are precursors of highly bioactive molecules, produced by the enzymatic (i.e.,cyclooxygenases and lipoxygenases) or non-enzymatic metabolism of α,β-unsaturated aldehydes (e.g., 4-hydroxynoneal and 4-hydroxyhexenal), cyclopentenone metabolites (e.g., isoprostanes and neuroprostanes), nitrated fatty acids (e.g., NO_2_-CLA, NO_2_-LA), and specialized pro-resolving mediators (e.g., lipoxins, resolvins and maresins) involved in the initiation, progression, and resolution of the inflammatory process [21]. The brain is particularly susceptible to oxidative stress because it has a high demand for oxygen, high concentrations of iron, and relatively low antioxidant capacity, as well as high content of PUFAs, which are source of oxygen radicals through lipid peroxidation [174,175]. Oxidation of membrane PUFAs alters the fluidity of membranes, damages membrane receptors structures, causes mitochondrial dysfunction, and deteriorates cellular components, thus contributing to neuronal degeneration and impaired neuronal communication [167].

Interestingly, it was observed that DHA from dietary sources is incorporated into mitochondrial and synaptosomal membranes in mouse brains [176]. Mitochondrial phospholipids are particularly rich in DHA that promotes mitochondrial biogenesis and modulates the expression of genes associated with energy metabolism and ATP production [177]. Alteration of the mitochondrial oxidative capacity is linked to brain aging and plays a crucial role in the onset and progression of neurodegenerative diseases, such as Alzheimer’s, Parkinson’s, Huntington’s disease, and ischemic stroke [159,178]. Interestingly, the ability of ω-3 PUFA to reverse inflammation, oxidative stress, and altered mitochondrial functions has been demonstrated as observed in animal models of obesity [179]. These data suggest that these FAs have a pivotal role in mitochondrial functions and efficiency.

**Figure 1:**
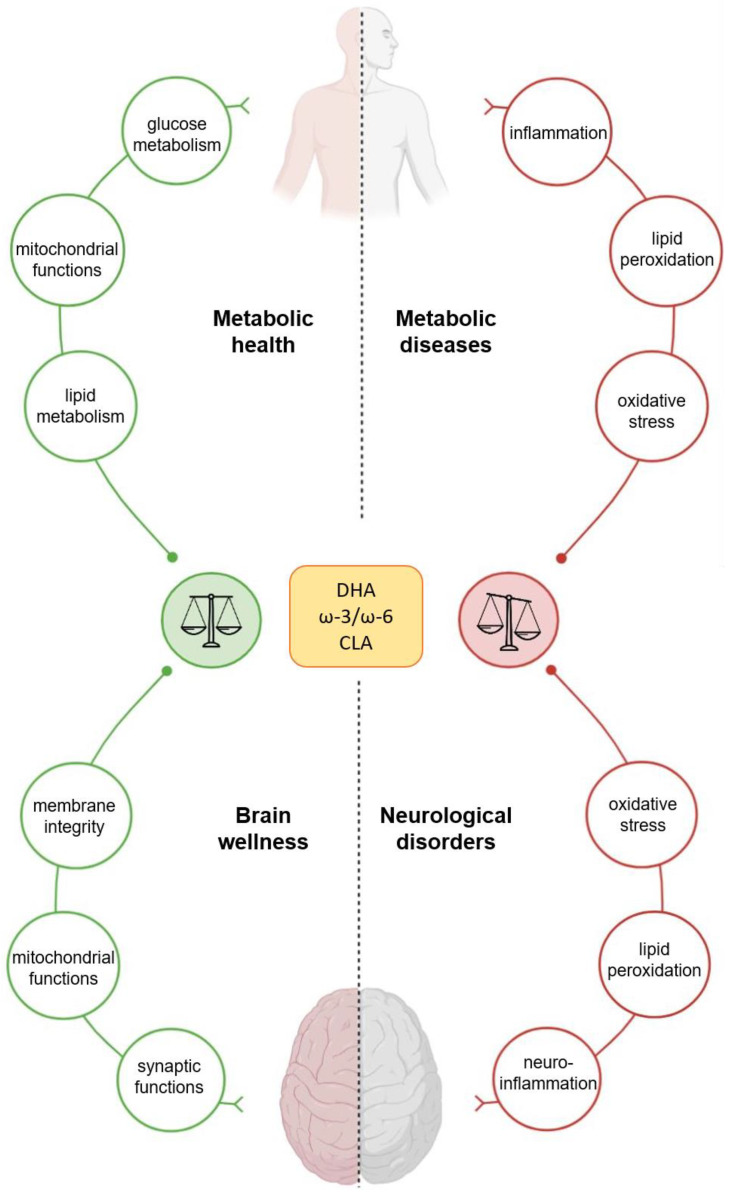
Effects of docosahexaenoic acid (DHA), conjugated linoleic acid (CLA), and ω-3/ω-6 on human wellness. The nutritional supply of DHA, CLA, and ω-3–ω-6 PUFAs has a great impact on human health, affecting the metabolism of peripheral organs and the brain’s functions.

CLA is able to cross the blood–brain barrier and be incorporated and metabolized in the brain, where it plays different roles [180,181]. Peculiarly, CLA has been shown to induce the biosynthesis of endogenous PPAR-α ligands, PEA and OEA, likely through a positive feedback mechanism where PPAR-α activation sustains its own cellular effects through ligand biosynthesis [182]. In addition to PPAR-α, PEA and OEA can activate, indirectly or directly, other receptors such as transient receptor potential vanilloid 1 (TRPV1) [183,184], which are also implicated in metabolism regulation and anti-inflammatory activity [185]. These data further extend CLA anti-neuroinflammatory actions, demonstrated in cultured astrocytes [186] and in vivo, where it has been shown that chronic dietary CLA intake reduces prostaglandin E2, decreases the activity of the endocannabinoid system, and inhibits angiogenesis in the mammalian brain [187,188,189]. In a murine model of auto-immune disease, it has been shown that the protective effect of CLA on age-dependent biochemical signs of neurodegeneration and depression are associated with the activation of Nrf2-mediated cytoprotective defences [190,191]. In addition, CLA protects against the disease-associated decline of synaptogenesis [190]. Moreover, RA has been shown to protect mouse cortical neurons from glutamate excitotoxicity [192]. Interestingly, the CLA supplementation of pregnant rats affects brain functions in offspring. Indeed, a maternal CLA-rich diet during gestation and lactation results in beneficial effects on neurodevelopment, improving learning and memory in the newborn rats [193] (Figure 1).

## 6. Conclusions

**Figure 2:**
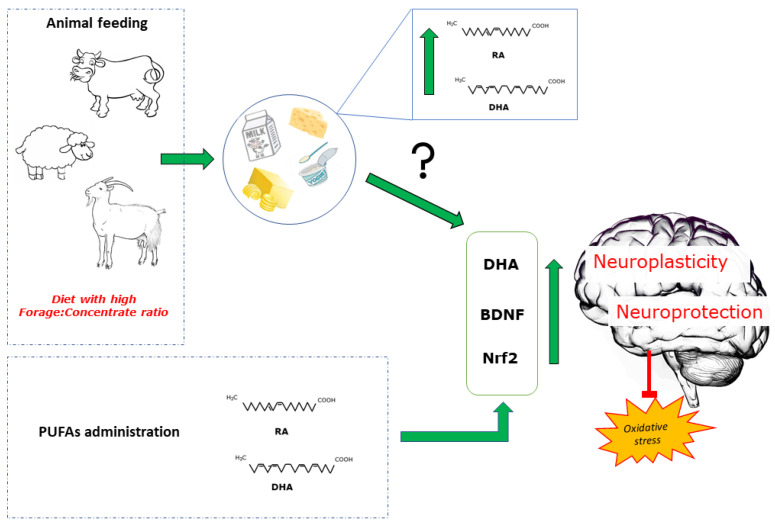
Animal feeding systems affect the profile of milk PUFAs (rumenic acid, RA; docosahexaenoic acid, DHA). Studies related to the effects of milk administration on metabolic and brain health yielded controversial results. Administration of pure PUFAs increases DHA brain content, stimulates brain-derived neurotrophic factor (BDNF) expression in several brain areas, and activates the nuclear erythroid-related factor 2 (Nrf2) in the brain. These factors participate in redox homeostasis and modulate inflammatory state, contributing to neuroplasticity and neuroprotection.

FAs composition of dairy products varies among different species, and it is strongly influenced by animal nutrition. Milk and dairy products greatly contribute to human daily intake of FAs that, beside their function as structural components of cells and metabolic substrate, also play a crucial role as signaling molecules. Peculiarly, a higher content of potentially beneficial FAs adds nutritional/functional value to dairy products. Numerous comparative evaluations have been conducted in different animal species, nutritional strategies have been developed, and increased commercial attention is accompanying “enriched” products. Unfortunately, there are very few in vivo data supporting their beneficial activities, and most of them have been obtained by using pure FAs (mainly ω-3 PUFAs), rather than FAs naturally incorporated into enriched milk/cheese, which may possess distinct biological activities compared to the pure FAs. Thus, although our review provides various evidence of the beneficial effects of dietary PUFAs, characteristic of milk and dairy products, on metabolic and neuronal functions, more preclinical and clinical studies are warranted to confirm and further investigate the effects of milk and dairy products on human health (Figure 2).

## Figures and Tables

**Table 1:** Milk fat and fatty acid profiles in ruminant and non-ruminant species (data from [37,38,39,40,41,42]).

	Cow	Buffalo	Sheep	Goat	Horse	Donkey
Fat (g/100 g)	3.3.–6.4	5.3–15.0	4.0–9.0	3.0–7.2	0.4–7.2	0.3–1.8
	% of total FAs					
SFAs	55.0–73.0	62.0–74.0	57.0–75.0	59.0–74.0	37.0–55.0	46.0–68.0
MUFAs	2.0–30.0	24.0–29.0	23.0–39.0	19.0–36.0	18.0–36.0	15.0–35.0
PUFAs	2.4–6.3	2.3–3.9	2.6–7.3	2.6–5.6	13.0–51.0	14.0–30.0
CLA	0.2–2.4	0.4–1.0	0.6–1.1	0.3–1.2	0.02–0.1	Trace
ω-6	1.2–3.0	1.74–2.0	1.6–3.6	1.9–4.3	3.6–20.3	6.0–15.2
ω-3	0.3–1.8	0.2–1.4	0.5–2.3	0.3–1.48	2.2 -31.2	4.0–16.3

SFAs, saturated fatty acids; MUFAs, mono-unsaturated fatty acids; PUFAs, polyunsaturated fatty acids, ω-3, omega-3; ω-6, omega-6.

**Table 2:** Summary of the main studies focused on the biological effects produced by the intake of RA-rich dairy products (data from [37,127,128,131,134,142,143,144,145,146,147,148,149,150]).

	Study	Treatment	Outcome
Animal models			
	Female growing pigs	RA-enriched butter	Undetectable effects on blood lipoproteins [142]
	Wistar rats	RA-enriched clarified butter	↑ Antioxidants [143]
	Wistar rats	RA-enriched butter	↑ HDL, Triacylglycerol [144]
	Mice fed high fat diet	Pasture dairy cream	↓ Inflammation, Triacylglycerol ↑ Protective cells in the gut [131]
	Wistar rats	RA-enriched butter	↑ PLA2 [145]
	Mouse model of chemically -induced (DSS) colitis	RA-enriched butter	↑ Nrf-2- mediated defenses ↓ Colitis signs [146]
	Wistar rats	RA-enriched butter	↑ Mitochondrial function [37]
	Wistar rats	RA-enriched butter	↓ Muscle inflammation, oxidative stress ↑ Mitochondrial function [128]
Clinical studies			
	Healthy middle-age subjects	Naturally enriched dairy products	Undetectable effects on blood lipoproteins [147]
	Healthy normal-weight and over-weight subjects	Naturally enriched cheese	↓ IL-6, Tnf-α [148]
	Hypercholesterolemic subjects	Naturally enriched cheese	↓ LDL [134]
	Healthy young subjects	Naturally enriched cheese	↑ IL-10 ↓ NFkB, Tnf-α, IL-2, IL-8 [149]
	Meta-analyses	CLA enriched food	↓ LDL, cholesterol [150]
	Healthy middle-age subjects	RA enriched cheese	↑ Highly unsaturated fatty acids in blood plasma [127]

RA, rumenic acid; HDL, high density lipoprotein; PLA2, phospholipase A2; DSS, dextran sulfate sodium; Nrf-2, nuclear factor E2-related factor 2; IL-6, interleukin-6; Tnf-α, tumor necrosis factor- α; LDL, low density lipoprotein; IL-10, interleukin-10; NFkB, nuclear factor kappa-beta; IL-2, interleukin-2; IL-8, interleukin-8; CLA, conjugated linoleic acid.

## Abbreviations

FAs	Fatty acids
LA	Linoleic Acid
CLA	Conjugated linoleic acids
PUFAs	Polyunsaturated fatty acids
SFAs	Saturated fatty acids
CNS	Central nervous system
AA	Arachidonic acid
EPA	Eicosapentaenoic acid
DHA	Docosahexaenoic acid
RA	Rumenic acid
ALA	α-linolenic acid
tVA	Trans-vaccenic acid
OEA	Oleoylethanolamide
PEA	Palmitoylethanolamide
PPAR	Peroxisome proliferators-activated receptors
BCFA	Branched chain fatty acids
OCFAs	Odd-chain fatty acids
OBCFAs	Odd-branched-chain fatty acids
BDNF	Brain derived neurotropic factor
Nrf	Nuclear factor erythroid 2-related factor 2
